# Two cases of polyorchidism: case report and literature review

**DOI:** 10.3389/fped.2025.1563191

**Published:** 2025-10-01

**Authors:** Jiaxiang Tang, Qi Liu, Zhifei Zhao, Hongting Lu

**Affiliations:** Qingdao University Affiliated Women and Children's Hospital, Qingdao, Shandong, China

**Keywords:** polyorchidism, children, diagnosis, treatment, iconography

## Abstract

**Objective:**

To explore the diagnosis, clinical presentation, and management of polyorchidism, aiming to enhance awareness of this rare condition.

**Methods:**

A retrospective analysis was conducted of two children diagnosed with polyorchidism at the Qingdao University Affiliated Women and Children's Hospital between December 2022 and January 2025. A comprehensive review of the relevant literature was also performed.

**Results:**

(1) Case 1: A 3-year and 9-month-old male presented with a palpable mass in the right scrotum, initially diagnosed as a right-sided inguinal hernia. Preoperative ultrasound suggested the mass might be a testicle. The mass was completely excised and sent for pathological examination, which confirmed the diagnosis of polyorchidism. Case 2: A 7-year-old male presented with a reducible mass in the left scrotum for 6 months and a history of phimosis. Initial diagnosis included left-sided inguinal hernia and phimosis. Preoperative ultrasound suggested a left inguinal hernia, with bilateral testicular asymmetry. The right testicle showed increased mobility, and an echoic mass was observed in the right scrotum, indicating the possibility of polyorchidism. The patient underwent laparoscopic high ligation of bilateral hernia sacs, excision of the right scrotal mass, and circumcision. Pathology confirmed the diagnosis of polyorchidism. Both testicles of the two children could be palpated in the scrotum, with normal texture and no tenderness. Both children were cured and discharged without complications. Follow-up showed no abnormalities. (2) A summary of the clinical features and treatment of polyorchidism was compiled from both this case series and previous reports.

**Conclusion:**

Polyorchidism is an extremely rare congenital anomaly of the male reproductive system. Pediatric surgeons and urologists should increase awareness of this condition to avoid misdiagnosis and delayed diagnosis.

## Introduction

Polyorchidism, a condition characterized by the presence of three or more testicles in males, is an extremely rare congenital anomaly of the urogenital system. The additional testicles are typically located within the scrotum but can also be found in the inguinal region or retroperitoneal space. The clinical presentation of polyorchidism is nonspecific and is often discovered incidentally during the management of conditions such as cryptorchidism, hydrocele, inguinal hernia, testicular torsion, or hypospadias (1, 2). In some cases, polyorchidism may present as a painless mass in the scrotal or inguinal area (3). We report two cases in which polyorchidism was discovered during the surgical treatment of inguinal hernia and confirmed through pathological diagnosis, followed by a review of the literature to raise awareness of this rare condition.

## Clinical case

### Case 1

**Figure 1:**
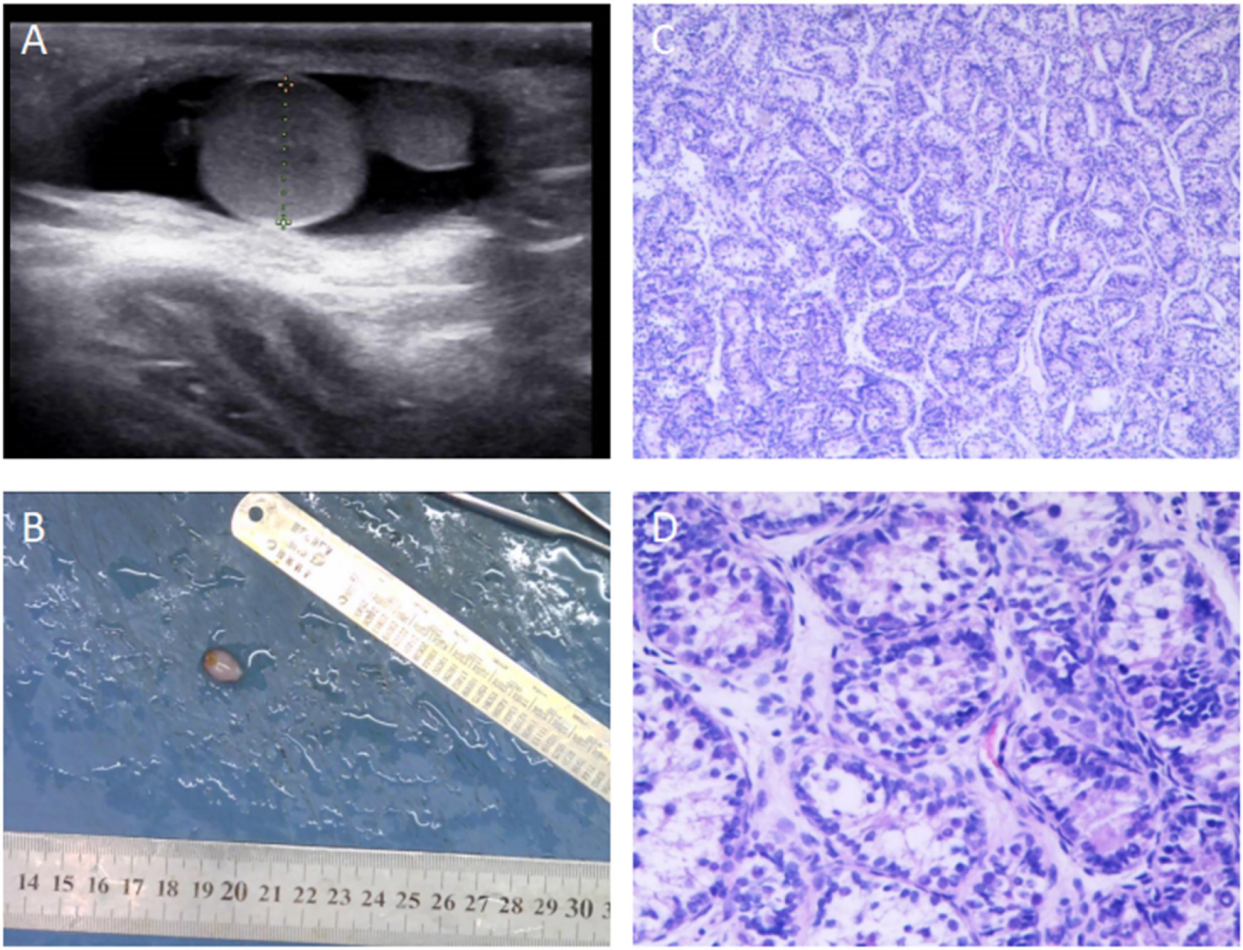
Preoperative Ultrasound and Postoperative Pathology of Polyorchidism in a Pediatric Patient. **(A)** Preoperative ultrasound image showing a hypoechoic mass in the right scrotum near the tail of the epididymis, measuring approximately 0.9 × 0.6 cm. The mass had clear borders, and the echogenicity was similar to that of testicular tissue. A fluid-filled area with a depth of 1.7 cm was also observed in the right scrotum, with good transmission. The longitudinal axis of the right testis was 1.04 cm, and the transverse diameter was 0.71 cm. **(B)** Macroscopic appearance of the excised specimen sent for pathological examination. **(C)** Postoperative pathological examination showing convoluted seminiferous tubules (HE ×100). **(D)** Postoperative pathological examination showing convoluted seminiferous tubules (HE ×200).

A 3-year and 9-month-old male presented on December 14, 2022, with a 6-month history of a mass in the right scrotum. The mass was reducible into the abdominal cavity, becoming more prominent during physical activity and disappearing when the child was at rest. The patient did not report any pain or discomfort and had no prior medical interventions. The patient's past medical history was unremarkable, with no known hereditary or infectious diseases, and no history of trauma or surgeries.On physical examination, the child was well-developed with normal genitalia. The right scrotum exhibited a soft mass measuring approximately 2.0 cm × 2.0 cm × 1.5 cm, which was non-tender and could be completely reduced into the abdomen. The transillumination test was negative. The testes were palpable bilaterally within the scrotum, with normal consistency and no tenderness.Preoperative ultrasound showed the left testis measuring 1.3 cm × 0.8 cm, and the right testis measuring 1.1 cm × 0.8 cm. Both testes appeared normal with well-defined borders and homogeneous echogenicity. In the right scrotum, a hypoechoic mass measuring approximately 0.9 cm × 0.6 cm was identified, with a well-defined margin and echogenicity resembling that of testicular tissue. A liquid dark area was also noted in the right scrotum, measuring 1.7 cm deep with good transmission. The diagnosis was initially made as a right-sided inguinal hernia and scrotal mass.The patient underwent laparoscopic high ligation of the hernia sac and excision of the scrotal mass. During surgery, a firm, well-defined mass measuring 0.7 cm × 0.6 cm × 0.5 cm was palpated at the lower pole of the right testis, and was found to be attached to the tunica vaginalis. The mass was excised, and pathological examination revealed a grayish-yellow nodular lesion measuring 1.0 cm × 0.3 cm × 0.3 cm, with a smooth surface and a grayish-white, slightly yellow cut surface. Microscopically, the mass consisted of convoluted seminiferous tubules surrounded by a white, membranous structure, confirming the diagnosis of polyorchidism (Figure 1). One month postoperatively, follow-up ultrasound showed normal testicular development without any significant findings. Six months later, the child had no clinical abnormalities, and scrotal ultrasound showed no significant changes.

### Case 2

**Figure 2:**
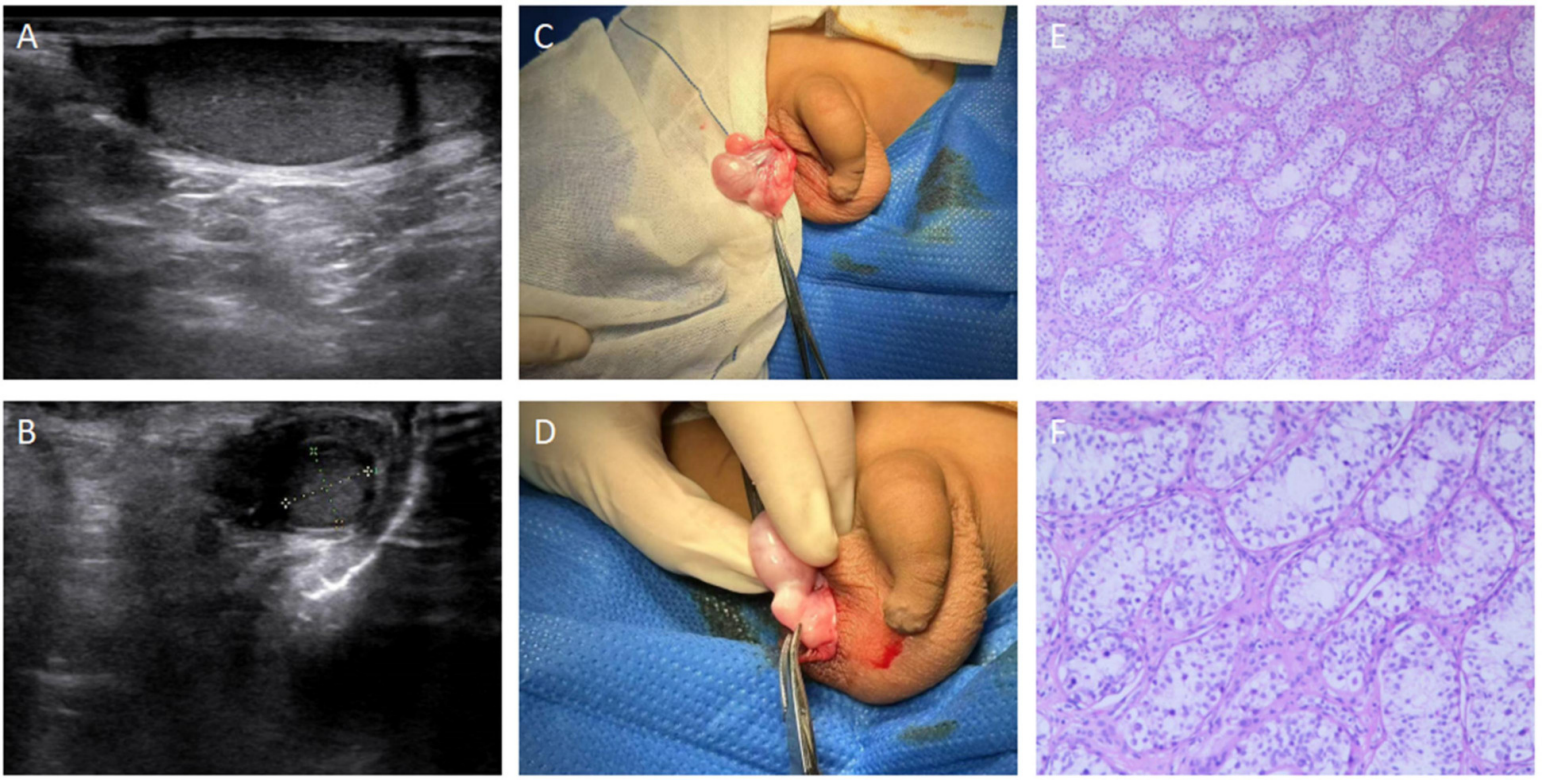
Preoperative Ultrasound, Intraoperative Photos, and Postoperative Pathology of Polyorchidism in a Pediatric Patient. **(A)** Preoperative ultrasound image showing a hypoechoic mass below the right testicle, measuring 0.6 × 0.5 cm. The echogenicity of the mass is similar to that of the testis, with clear borders and regular shape. The longitudinal axis of the right testis is 1.8 cm, and the transverse diameter is 1.0 cm. **(B)** Preoperative ultrasound image showing an additional right testis with a longitudinal axis of 0.9 cm and a transverse diameter of 0.6 cm. **(C,D)** Intraoperative photos showing the location of the additional testis. **(E)** Postoperative pathological examination showing convoluted seminiferous tubules (HE ×100). **(F)** Postoperative pathological examination showing convoluted seminiferous tubules (HE ×200).

A 7-year-old male patient was admitted to the hospital on January 5, 2025, due to a reversible mass in the left scrotum for half a year and a narrow prepuce for half a year. The mass was reducible into the abdominal cavity without impaction or tenderness. Six months ago, the family of the child found that the foreskin of the child was narrow, and he had 2 times of redness, swelling, and pain, which improved after topical drug treatment. He had no dysuria, urinary frequency, or urgency; he had no nausea, vomiting, or fever. His past medical history included asthma and a febrile seizure. Family history was not affected, and there was no history of trauma or previous surgery. On physical examination, the patient's external genitalia were normal, but the prepuce was narrow and unable to retract to expose the glans and coronal sulcus. A mass was palpated in the left inguinal region extending into the scrotum, measuring approximately 3 × 3 × 2 cm. The mass was soft, non-tender, and could be reduced into the abdominal cavity, with a negative transillumination test. Both testicles were palpable within the scrotum, and a well-defined, non-tender mass measuring 0.8 × 0.7 cm was palpated just below the right testicle. Preoperative ultrasound revealed a heterogeneous hypoechoic mass in the left inguinal canal, measuring approximately 4.5 × 1.7 cm, with the base connected to the abdominal cavity. The left testis measured 2.4 × 1.1 cm with clear borders, normal shape, and homogeneous echogenicity. The right testis measured 1.8 × 1.0 cm, with similar findings, and increased mobility was observed. Below the right testis, an additional hypoechoic mass measuring approximately 0.6 × 0.5 cm was observed, with similar echogenicity to the testis. The right and left epididymides showed no significant abnormalities, and the bilateral spermatic veins were not dilated. The preoperative diagnosis included: Left-sided inguinal hernia, Phimosis, Right scrotal mass, possible polyorchidism. The patient underwent laparoscopic high ligation of the hernia sacs, excision of the right scrotal mass, and circumcision. Intraoperatively, both internal inguinal rings were found to be patent, and laparoscopic high ligation of the hernia sacs was performed. A circumcision was also done. A 2 cm transverse incision was made in the lower third of the right scrotum. The skin, dartos, and tunica vaginalis were carefully dissected to expose a well-defined, firm, round mass measuring 0.9 × 0.7 cm, which was attached to the lower pole of the right testis. The mass was excised using blunt and sharp dissection, and hemostasis was achieved. The tunica vaginalis was closed using absorbable sutures, and the wound was closed in layers. The excised right scrotal mass was sent for pathological examination. Postoperative pathology revealed a grayish-white nodular lesion, measuring 1.0 × 1.0 × 0.6 cm, with a smooth surface and slightly firm consistency. The cut surface was grayish-white with a glossy appearance. Pathological examination confirmed the presence of convoluted seminiferous tubules, consistent with polyorchidism (Figure 2). One week postoperatively, follow-up ultrasound showed no significant abnormalities in either testis or epididymis. The scrotum was healing well, and no complications were noted.

## Literature review

### Search method

The relevant literature reports in the PubMed database up to January 2025 were retrieved using the search terms “polyorchidism” and “supernumerary testis”. A total of 265 records of polyorchidism from 1980 to 2025 were found in the PubMed database, among which approximately 160 cases were confirmed by pathological examination. The Wanfang Database, VIP Database, and China National Knowledge Infrastructure (CNKI) were searched for literature on polyorchidism from 1980 to 2025 using the search term “多睾症”. After removing duplicate records, a total of 90 cases of polyorchidism reported in China were retrieved, of which 59 cases were confirmed by pathological examination.

### Eligibility assessment

The information of patients diagnosed with polyorchidism was extracted based on the following inclusion and exclusion criteria. Inclusion criteria: (1) Diagnosed as “polyorchidism” and “supernumerary testis”; (2) Diagnosed between 1980 and 2025. Exclusion criteria: (1) Review articles and conference abstracts; (2) Polyorchidism in animals; (3) Incomplete data.

Among these cases, triorchidism is the most common form of polyorchidism, although extremely rare instances of quadriorchidism and pentaorchidism have been reported. In 65% of cases, polyorchidism occurs on the left side. Approximately 76% of the additional testes are located within the scrotum, while 24% are outside the scrotum. Among those located outside the scrotum, 87% are in the inguinal region, and 13% are in the abdominal cavity. About 80% of additional testes are identified through imaging studies, while the remaining 20% are discovered incidentally during surgery. Imaging techniques, such as ultrasound and MRI, have proven useful in early detection of additional testes, aiding in the diagnosis of polyorchidism.

In terms of associated conditions, 17% of polyorchidism cases are concomitant with ipsilateral inguinal hernia, 15% with cryptorchidism, 7% with hydrocele, 6% with testicular torsion, and 4% with testicular cancer detected in the additional testis upon pathological examination. About 40% of polyorchidism cases are incidentally discovered during the evaluation of unrelated conditions, such as inguinal hernia, hydrocele, or testicular torsion.

## Discussion

The etiology of polyorchidism is closely related to abnormal transverse splitting of the genital ridge before the 8th week of pregnancy. During embryonic development in the 6th week, the testicular anlage begins to form from the primitive gonadal ridge in the mesoderm, migrating toward the mesonephros. By the 8th week, the development of the epididymis and vas deferens starts from the mesonephric ducts. If abnormal splitting of the genital ridge occurs during this period, or if there is an anomaly in the development of the epididymis and vas deferens, it can lead to polyorchidism (4). Furthermore, bilobed testes, a rare form of polyorchidism, are likely caused by incomplete splitting of the genital ridge (5).

In approximately 4% of polyorchidism cases, a testicular tumor is discovered concurrently. Imaging studies can help differentiate polyorchidism from malignant lesions (1). MRI imaging of normal adult testes shows a high signal on T2 and low signal on T1, with an elliptical structure, uniform in appearance, and limited diffusion, surrounded by the low signal white membrane on both T1 and T2 (1). In polyorchidism, the additional testis has a signal intensity similar to that of a normal testis (6).

The incidence of testicular torsion in the general population is approximately 0.025% (7), but the risk is higher in polyorchidism patients, estimated at 0.25%. Bergholz et al. (8) reported that up to 15% of polyorchidism cases were diagnosed with ipsilateral testicular torsion. Additionally, the risk of testicular cancer is higher in polyorchidism patients, with a malignancy rate in additional testes as high as 6%, compared to normal testes (9). Early in pediatric urology practice, the risks of testicular torsion and potential malignancy in additional testes were major concerns. Therefore, surgical excision was often employed as the treatment strategy (10, 11). However, with advances in imaging technology, conservative management and observation have become viable options for patients who do not require surgery. Surgical exploration and pathological examination have shown that 50%–67% of additional testes contain functional tissue (10). Based on this, conservative treatment with regular imaging follow-up is also considered an acceptable approach. Consequently, there is an increasing tendency in pediatric urology to retain additional testes, provided there are no clear clinical symptoms or signs of malignancy.

The classification system of polyorchidism proposed by Bergholz (8) in 2009 mainly relies on whether the supernumerary testis has a vas deferens drainage and its anatomical relationship with the main testis, and is divided into two major types and several subtypes. Type A: In this type, the supernumerary testis has a vas deferens drainage. According to whether the epididymis and vas deferens are independent, it is further divided into: A1 subtype: The supernumerary testis has a completely independent epididymis and independent vas deferens, and has no anatomical continuity with the main testis. A2 subtype: The supernumerary testis has an independent epididymis, but its vas deferens is shared with the main testis. A3 subtype: The supernumerary testis and the main testis share the epididymis and vas deferens, and their anatomical relationship is close. A4 subtype: The supernumerary testis has an independent vas deferens, but the epididymis is partially or completely shared with the main testis. Type B: In this type, the supernumerary testis lacks a complete vas deferens system and usually has no reproductive function. According to whether the epididymis exists, it is divided into: B1 subtype: The supernumerary testis has an epididymis structure, but no vas deferens connection. B2 subtype: The supernumerary testis only contains testicular parenchyma, and has neither epididymis nor vas deferens. The Bergholz classification system is helpful for intraoperative anatomical identification and determination of individualized surgical plans. The two cases of polyorchidism in this article may belong to the A3 subtype, where the supernumerary testis and the main testis share the epididymis and vas deferens, and are both covered by the tunica vaginalis. In both polyrchidismal surgeries, we performed delicate manipulations to avoid damaging the structures involved. At the end of the operation, the main testicle is probed to have a clear vas deferens and testicular blood vessels.

**Figure 3:**
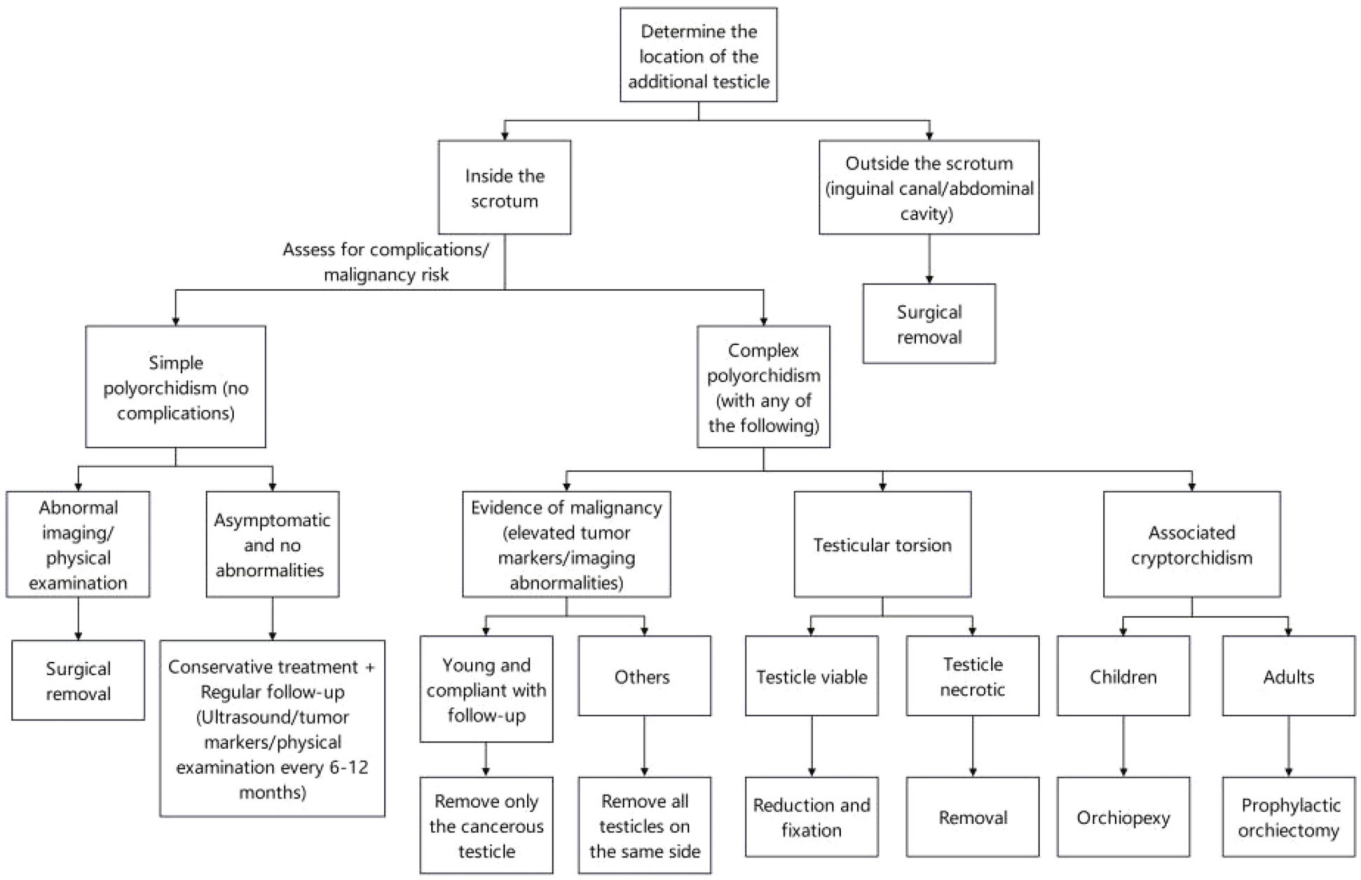
The selection of treatment options for polyorchidism.

In clinical practice, the decision to surgically remove an additional testis is generally based on two considerations: whether the testis has potential fertility and whether it carries a risk of malignancy. Bergholz et al. (8) in their retrospective study found that malignant additional testes were more commonly located in the abdominal or inguinal regions. For additional testes located outside the scrotum, surgical removal is typically recommended. However, for additional testes located within the scrotum, conservative treatment with regular follow-up is often considered unless there are significant clinical symptoms or signs of malignancy. Khedis et al. (12) suggested that in simple polyorchidism, where there is no evidence of malignancy or associated abnormalities, and when the additional testis is located within the scrotum, conservative management may be appropriate. In complex polyorchidism, if malignancy is suspected, the affected testis, including the main testis, should be excised. If the patient is young and cooperative, only the suspected malignant testis may be removed. In cases of testicular torsion, if the testis is viable, it can be detorsioned and fixed; if necrotic, it should be excised. In cases of cryptorchidism, pediatric patients may undergo orchidopexy, while adult patients may require orchiectomy to prevent malignancy. In instances of uncertainty, intra-operative frozen-section analysis could have been employed to support a more informed decision. The issue of removing healthy supernumerary testicular tissue seems worthy of consideration. In cases of polyorchidism with functional testicular tissue, whether to remove healthy testicular tissue requires careful consideration. We tend to adopt a conservative treatment approach for such cases. Similarly, there is literature (12) supporting that a conservative treatment approach is feasible in such cases, which can protect the patient's fertility and endocrine function. However, conservative treatment may increase the risk of testicular torsion and other complications, which requires a balance in clinical treatment decisions (Figure 3). Additionally, orchiopexy can effectively reduce the risk of torsion and address some of the concerns of conservative treatment, but the surgical trauma and potential benefits need to be weighed. When dealing with polyorchidism, doctors need to follow relevant medical regulations and guidelines to ensure the legality and standardization of treatment. At the same time, sufficient attention should be given to aspects such as informed consent and privacy protection for patients to avoid possible legal disputes.

## Conclusion

Polyorchidism is a rare congenital anomaly of the male reproductive system, and its exact etiology and pathogenesis remain incompletely understood. It is often discovered incidentally in association with other conditions, but polyorchidism should always be considered in the differential diagnosis of scrotal or inguinal masses. Advances in modern imaging techniques have greatly facilitated the diagnosis of polyorchidism. Treatment strategies should consider the child's age, location of the additional testis, fertility potential, risk of malignancy, and the preferences of the patient and their family.

## Data Availability

The original contributions presented in the study are included in the article/Supplementary Material, further inquiries can be directed to the corresponding author.
